# Editorial: Cell Death in Cyanobacteria

**DOI:** 10.3389/fmicb.2021.760549

**Published:** 2021-10-04

**Authors:** Piotr Rzymski, María Victoria Martin, Leda Giannuzzi

**Affiliations:** ^1^Department of Environmental Medicine, Poznan University of Medical Sciences, Poznań, Poland; ^2^Integrated Science Association (ISA), Universal Scientific, Education and Research Network (USERN), Poznań, Poland; ^3^Instituto de Investigaciones en Biodiversidad y Biotecnología (INBIOTEC-CONICET), Fundación para Investigaciones Biológicas Aplicadas (CIB-FIBA), Mar del Plata, Argentina; ^4^Centro de Investigación y Desarrollo en Criotecnología de Alimentos, Consejo Nacional de Investigaciones Científicas y Tecnológicas, Universidad Nacional de La Plata, La Plata, Argentina; ^5^Área de Toxicología General, Facultad de Ciencias Exactas, Universidad Nacional de La Plata, La Plata, Argentina

**Keywords:** cyanobacteria, regulated cell death, orthocaspases, polyphenols, reactive oxygen species, toxin-antitoxin module

Cyanobacteria are photosynthetic prokaryotic microorganisms with a wide geographic distribution and adapted to different aquatic and terrestrial environments. High temperature, sunlight exposure and eutrophication can lead to cyanobacterial blooms, representing global ecological and human health issues. Numerous works have focused on conditions promoting the massive proliferation of cyanobacteria, while cyanobacterial demise has scarcely been investigated. Moreover, unlike cell death in eukaryotic cells, little is known about regulated cell death in cyanobacteria. Different investigations reported various types of cyanobacterial death induced under several stressful conditions (Berman-Frank et al., 2004; Hu and Rzymski, 2019). This caused a need for sound classification of cyanobacterial death modes and their further studies. This editorial summarizes the contributions to the Frontiers Research Topic entitled “*Cell Death in Cyanobacteria*” for which we had the honor to serve the editors and which gathers publications that present the state-of-the-art knowledge of regulated cell death in these oxygenic photosynthetic microorganisms.

This Research Topic aimed to explore recent developments in this area with a focus on (1) The evolution, mechanism, genetic machinery, and biochemical activities associated with cyanobacterial regulated cell death pathways, (2) The impact and role of cyanobacterial death on phytoplankton dynamics and the microbial loop and (3) Methods, experimental systems and approaches to study cell death in laboratory and field.

In this topic, Aguilera et al. analyzed the state of knowledge in the field of cyanobacterial cell death and discussed the terminology adopted in the field. The authors propose a unified criterion for the definition of accidental, regulated, and programmed forms of cell death in cyanobacteria based on molecular, biochemical, and morphological aspects following the directions of Nomenclature Committee on Cell Death (NCCD). This contribution provides a guide to standardize the nomenclature related to this topic in a precise and consistent manner, which will facilitate further ecological, evolutionary and applied research in the field of cyanobacterial cell death.

Giannuzzi et al. analyzed cell death in *Microcystis aeruginosa* by means of a stochastic dose-response model using hydrogen peroxide and flow cytometry to study cell viability and characterize transitional cell death phenotypes. A logistic cumulative distribution function was applied to calculate the half-maximal effective concentration (EC_50_). The authors conclude that EC_50_, the stochastic model and the multidimensional transitional phenotypic analysis of single cells contribute to further characterization of cell death pathways in cyanobacteria.

Bhattacharjee et al. performed a comprehensive bioinformatics study describing the distribution, diversity, abundance, domain configuration, and phylogeny of 473 putative cyanobacterial orthocaspases among 62% of the cyanobacterial strains subsumed within all taxonomic orders.

Klemenčič et al. investigated the possible interaction between *M. aeruginosa* PCC 7806 orthocaspase 1 (MaOC1) and the toxin-antitoxin module. The MaOC1 was able to cleave the antitoxin so that it could no longer block the toxin activity. The most likely target of the protease was the C-terminus of the antitoxin with two sections of basic amino acid residues. *E*. *coli* cells in which MaOC1 was expressed simultaneously with the toxin-antitoxin pair were unable to grow. In contrast, no effect on cell growth was found when using a proteolytically inactive MaOC1 mutant. The authors showed that the orthocaspase MaOC1, a cysteine protease encoded adjacent to the TA pair on the *M*. *aeruginosa* genome, cleaves the antitoxin in its free form but not when in complex with the toxin, thereby regulating the availability of the toxin in the cell. This is the first case of a cysteine protease that regulates the activity of a toxin-antitoxin module since all currently known activating proteases are of the serine type.

Huang et al. studied sensitive response parameters (including chlorophyll fluorescence parameters) of *M*. *aeruginosa* under the stress of different polyphenols individually and their combination. The combined effects on *M. aeruginosa* were investigated based on the most sensitive parameter and cell densities. The results show that increasing the ratio of high toxic polyphenols would not enhance the allelopathic effects, and the property, proportion and concentrations of polyphenols played an important role in the combined effects. Compared with cell densities, NPQ (light-induced photoprotection through thermal dissipation of energy in the antenna system) was a more suitable parameter for evaluating indicators in polyphenols' combined effects on *M*. *aeruginosa*. These results could provide a method to screen the allelochemicals of polyphenols inhibiting cyanobacteria and improve the inhibitory effects by different polyphenols combine.

Lema et al. evaluated the participation of a pseudo-orthocaspase (SyOC), a prokaryotic caspase homolog that lacks the p10 domain, in oxidative stress in the cyanobacterium *Synechocystis* sp. PCC 6803. To study the *in vivo* impact of this pseudo-protease during oxidative stress, the authors controlled its gene expression during exposure to H_2_O_2_ using RT-qPCR. A knock-out mutant was designed that lacked the pseudo-orthocaspase gene and its survival and growth rates were compared with those of wild-type cells, as well as with their proteome. SyOC deletion led to cells with greater tolerance to oxidative stress, suggesting that this protein may be involved in a pro-death pathway.

Rzymski et al. explored the effect of the extracts of *Daphnia magna* and *Daphnia cucullata* on the growth, intracellular ROS content, lipid peroxidation, programmed cell death (PCD), MC-LR release, and bound exopolysaccharide (EPS) level in *M*. *aeruginosa* during 7 days of exposure. The extracts of both daphnids induced dose-dependent growth inhibition, increase in ROS levels, lipid peroxidation, and PCD. The release of microcystin-LR (MC-LR) and an increase in the bound EPS fraction were observed in treated cultures. Generally, the greatest effects were found under the influence of extracts of *D*. *magna*, a larger daphnid with a higher feeding rate and grazing pressure on *Microcystis*. A potential cascade of events in *Microcystis* cells under the presence of daphnid-derived molecules suggests that this cyanobacterium may have a specific potential to sense the presence of grazers and initiate a response that is advantageous for population survival. The study indicates that grazer presence can potentially trigger a series of events in the *Microcystis* population, with cells undergoing oxidative stress-induced PCD associated with MC-LR release and increased EPS production by intact cells.

Franklin provided a review explaining how significant is the role of different forms of cell death in the cyanobacterial population dynamics. As explained, the evolutionary processes can account for an intrinsic form of cyanobacterial cell death, but the role of a wide range of biotic interactions cannot be disregarded. The paper highlights the need for further research, focusing not only on *Microcystis*, and encompassing high-resolution studies of cyanobacteria physiological state (mortality and oxidative stress, using consistent methods) in natural populations over the different seasons and bloom cycles, as well as culture studies (using fresh isolations and semi-natural continuous culture methods) investigating the performance of the methods available to characterize cell death, including biomarkers, thought to be associated with internal pathways, and oxidative stress.

In summary, this Frontiers topic outlined the first guide to standardize the nomenclature related to different types of cyanobacterial cell death, introduced new methodologies to study it, and provided insights into its molecular, biochemical, and evolutionary perspectives. Further research is required, particularly regarding the regulated cell death in cyanobacteria, its pathways and triggers. It is also essential to reaffirm the physiological relevance of laboratory studies using field investigations.

As editors of this Topic Collection, we express our gratitude to all the authors for their contributions and the reviewers for their thoughtful comments. We also thank Dr. Anabella Aguilera for her contribution to designing and organizing this editorial project. We are confident that this collection of papers advances our understanding of death in cyanobacteria and stimulates further, more in-depth studies on this phenomenon. Its exploration is essential for understanding cyanobacterial ecology, control of cyanobacterial blooms, and the fields of applied phycology and biotechnology.

## Author Contributions

This editorial was written jointly by all authors.

## Conflict of Interest

The authors declare that the research was conducted in the absence of any commercial or financial relationships that could be construed as a potential conflict of interest.

## Publisher's Note

All claims expressed in this article are solely those of the authors and do not necessarily represent those of their affiliated organizations, or those of the publisher, the editors and the reviewers. Any product that may be evaluated in this article, or claim that may be made by its manufacturer, is not guaranteed or endorsed by the publisher.
